# Occupational characteristics and disability-free survival after retirement age: an exploratory analysis from the ASPREE study

**DOI:** 10.3389/fpubh.2023.1191343

**Published:** 2023-12-15

**Authors:** Sheikh M. Alif, Geza P. Benke, Kathlyn J. Ronaldson, Karen Walker-Bone, Robyn L. Woods, Cammie Tran, Lawrence J. Beilin, Andrew M. Tonkin, Alice J. Owen, John J. McNeil

**Affiliations:** ^1^School of Public Health and Preventive Medicine, Monash University, Melbourne, VIC, Australia; ^2^School of Health Sciences, The University of Melbourne, Melbourne, VIC, Australia; ^3^Institute of Health and Wellbeing, Federation University Australia, Berwick, VIC, Australia; ^4^School of Medicine, The University of Western Australia, Perth, WA, Australia

**Keywords:** occupational characteristics, job exposure matrix, disability-free survival, mortality, older adult

## Abstract

**Background:**

Certain occupational characteristics have been linked with poor health and reduced longevity. However, the association between occupational characteristics and survival free of disability in a post-retirement age group has not been investigated.

**Methods:**

We investigated outcomes in 12,215 healthy older Australian adults in the Aspirin in Reducing Events in the Elderly (ASPREE) and ASPREE Longitudinal Study of Older Persons (ALSOP) sub-study. The ISCO-88 major occupational groups, settings, and activity levels were assigned based on free-text job descriptions. The Finnish Job Exposure Matrix was used to assign occupational characteristics to the three longest-held jobs. The primary endpoint, disability-free survival, was defined as a composite measure of death, dementia, or persistent physical disability. The endpoint of all-cause mortality was analyzed separately. Because of multiple exploratory analyses, only those associations with a two-sided value of p less than 0.005 were considered statistically significant. Cox proportional hazard models were used to calculate adjusted hazard ratios.

**Results:**

Having worked in an ‘elementary’ occupation was associated with a reduction in disability-free survival. A specific impact on disability-free survival was observed among those whose work had involved high accident risk and adverse social climate. No significant relationship was identified with those previously exposed to sedentary work, vigorous physical activity, work primarily outdoors, or a range of other occupational characteristics. All-cause mortality was not increased among any of the occupational groups.

**Conclusion:**

This exploratory study found a reduction in disability-free survival among people who worked in ‘elementary’ occupations, with specific risks associated with occupations involving high accident risks and adverse social climate.

## Introduction

Differences between occupations and their cumulative effects during working life are thought to contribute to variations in the incidence and mortality associated with several chronic diseases of aging (1). The Whitehall study of British civil servants was the first to demonstrate marked differences between mortality among individuals from different positions in the social hierarchy of occupations (2, 3). Higher mortality from cardiovascular diseases and a range of other causes of death occurred among those with the lowest-status occupations compared to those with higher managerial jobs although the extent to which this could be attributed to occupational, environmental, genetic, individual socio-economic, or lifestyle factors was unclear. Since then, many other studies have reported a relationship between various occupational characteristics and exposures and increased ill health and mortality later in life (4–6).

Most studies of the impact of occupations on future health have focused on mortality or on the incidence of specific diseases (5, 7–9). However, with many societies experiencing rapid aging of their populations, another social and economic imperative is to maintain an individual’s independence as long as possible, thereby reducing the need for institutional care. Disability-free survival, defined as survival free of severe physical disability or dementia has been proposed as a surrogate measure of independence, of particular relevance to studies of older people (10). To date, there is little information available about the impact of occupational factors on this important measure of population health.

The Aspirin in Reducing Events in the Elderly (ASPREE) study was a large, randomized clinical trial of low-dose aspirin and placebo in which disability-free survival was the primary outcome. Australian participants were healthy adults aged ≥70 years when recruited in 2010–2014 (11). Within a sub-study, an abbreviated occupational history, which included jobs held between approximately 1950 and 2009, was obtained. This analysis aims to determine the relationship between occupational characteristics and disability-free survival and mortality in healthy older adults. Understanding this relationship may help improve health outcomes and quality of life for older adults after retirement and inform public health policies and intervention strategies to promote healthy aging.

## Methods

### Study design and population

The detailed methodology of the ASPREE trial including its recruitment and primary endpoints has been reported (11). Participants enrolled were aged ≥70 years and were required to fulfill the eligibility criteria, which included the absence of previously diagnosed cardiovascular disease, dementia, physical disability, or any other illnesses likely to limit survival to less than 5 years. Shortly after enrolment, Australian ASPREE participants were invited to enroll in the ASPREE Longitudinal Study of Older Persons (ALSOP) sub-study which consisted of baseline and follow-up questionnaires (12). The information requested at baseline included information about occupational, environmental, lifestyle, behavioral, social, and economic factors. Ethical approval was obtained from the Monash University Ethics Committee (project numbers CF11/1100 and CF11/1935), and written informed consent was obtained from all participants.

### Occupational data coding and classification

A total of 12,498 participants provided information on up to three jobs that they had held for the longest duration during their working life. Information requested included the job title, employer industry, task descriptions, and years worked (12). These occupational histories were coded according to the four-digit International Standard of Occupations (ISCO-88) classification (13).

In the initial analysis, participants were categorized into nine main groups based only on the first digit of ISCO-88 codes. This initial digit indicates the primary occupational group for each participant among all potential occupations in the population. These occupational groups are (1) legislators, senior officials, and managers, (2) professionals, (3) technicians and associate professionals, (4) clerks, (5) service workers and shop and market sales workers, (6) skilled agricultural and fishery workers, (7) craft and related trades workers, (8) plant and machine operators and assemblers, and (9) elementary occupations (further details in Supplementary Table S1).

The ISCO-88 codes were converted to Finnish O-codes, and the Finnish Job Exposure Matrix (FINJEM) (up to 2007–09) was used to define occupational characteristics (14), as previously applied in the Australian population (15). For each job code, FINJEM indicates the likelihood that the job did or did not entail a particular exposure or characteristic. For these analyses, FINJEM was used to determine psychological, physical, and ergonomic exposures (definitions in Supplementary Table S2) (16). To simplify the analyses FINJEM exposures were categorized into dichotomous variables, ever-exposed vs. never-exposed (more detail in Supplementary Methods).

Based on job titles, the likely occupational setting was designated as indoors, outdoors, or combined indoor/outdoor. Additionally, the likely physical demands of the job were classified as mostly sitting or standing, involving moderate physical activity or vigorous physical activity. This classification was undertaken by a team experienced in occupational coding and verified by an expert occupational hygienist (GB).

### Endpoints

Participants were recruited from March 2010 to December 2014 and followed up for a mean of 6.3 (SD 1.8) years during the ASPREE trial and ASPREE-XT (extension). The primary endpoint, disability-free survival (DFS), was defined as the time to the first occurrence of death, dementia, or persistent physical disability (17). Physical disability was assessed for 6 months and was defined as a persistent loss of one of the six Katz Activities of Daily Living or admission to nursing home care because of physical disability. The presence of dementia was determined using the fourth edition of the Diagnostic and Statistical Manual of Mental Disorders by a panel of expert adjudicators (11, 17). Death was a secondary endpoint, identified during the trial period from health records, death notification from close contacts, and by linkage to the Australian National Death Index (17). The details of the schedule of health measures are listed in Supplementary Table S3 in the Online.

### Statistical analyses

Cox proportional hazards regression models with time-to-event analysis were used to compare the occupational setting, FINJEM exposures, and activity level with health outcomes. Hazard ratios (HRs) and 95% confidence intervals (CIs) were calculated, and participants were censored at the time of the event of interest. The proportional hazards assumption was checked using Schoenfeld residuals and found to be appropriate. A competing risk model was used with censoring at the time of death to develop the cumulative incidence plots.

The model was adjusted for age, sex, and smoking. Some of our analyses were further stratified by sex. Specific sensitivity analyses were carried out to account for education as additional confounding variables. With additional analyses, the hazard ratios were further adjusted for confounders such as alcohol use, body mass index, and hypertension. Cell sizes of less than five cases were not reported in the analyses, and to account for the multiple analyses, a two-sided value of p of <0.005 was used as the cutoff for statistical significance. All the analyses were conducted using Stata version 18 (StataCorp, College Station, TX).

## Results

**Figure 1:**
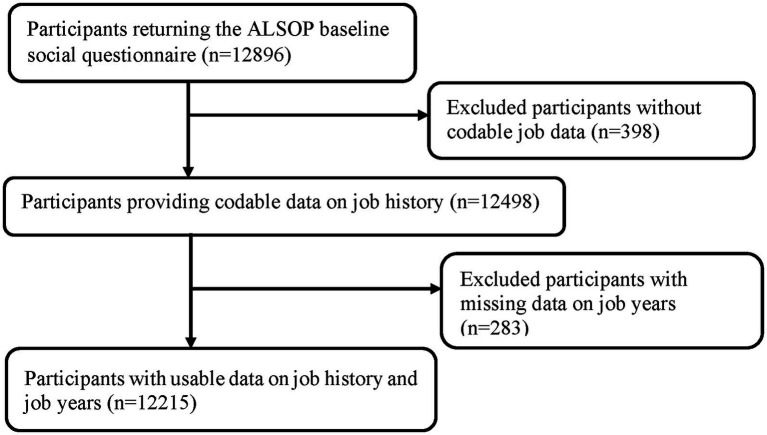
Flowchart illustrating the identification of the study population.

**Figure 2:**
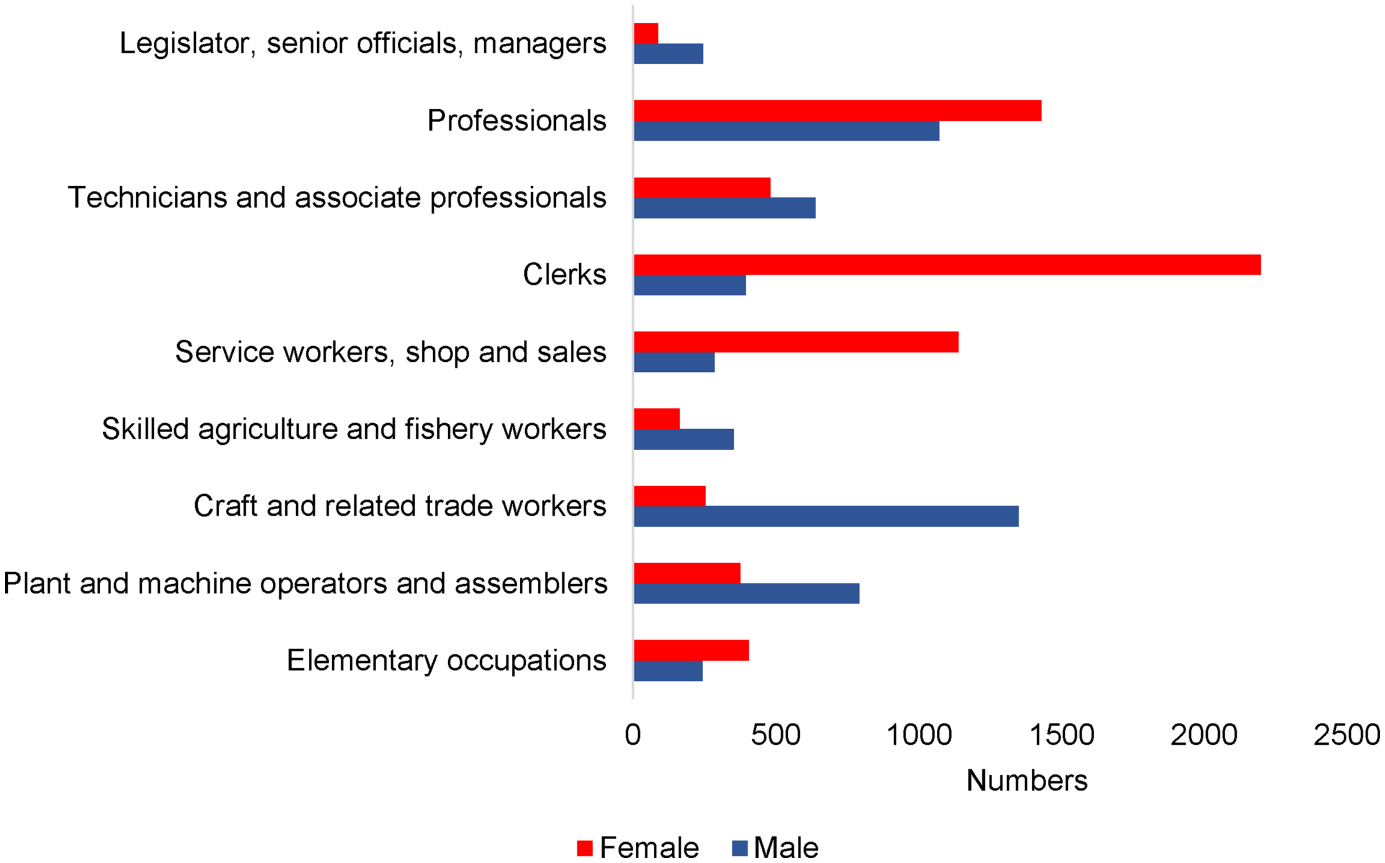
ISCO-88 major occupational groups by sex.

A total of 12,215 participants who provided work histories were included in this analysis (Figure 1). Table 1 shows their baseline characteristics. The predominant occupations for men were craft and trade workers (*n* = 1,349), followed by professionals (*n* = 1,072) and machine operators (*n* = 791). Among women, the predominant ISCO-88 occupations were clerks (*n* = 2,197), professionals (*n* = 1,429), and service workers or retail salespersons (*n* = 1,140; Figure 2).

**Table 1:** Baseline characteristics of ALSOP cohort stratified by occupational activity.

Study characteristics	Men, 5,569 (45.6%)	Women, 6,646 (54.4%)
Age, year (mean, SD)	75.07 (4.32)	75.21 (4.26)
Education, (%)		
<12 years education	2,498 (44.9)	3,333 (50.2)
≥12 years education	3,071 (55.1)	3,313 (49.9)
Body mass index, kg/m^2^, mean (SD)	27.85 (3.76)	27.98 (5.02)
Waist circumference, cm, mean (SD)	101.74 (10.48)	92.87 (12.53)
Smoking history *n* (column %)		
Never	2,389 (42.9)	4,421 (66.5)
Current	192 (3.5)	149 (2.2)
Former	2,988 (53.7)	2076 (31.2)
Alcohol users, *n* (%)		
Never	469 (8.4)	1,403 (21.1)
Current	4,796 (86.1)	4,979 (74.9)
Former	304 (5.5)	264 (4.0)
Diabetes, (%)	644 (11.6)	514 (7.7)
Hypertension, (%)	4,187 (75.2)	4,871 (73.3)
Paid employment, (%)		
No paid employment	46 (0.8)	34 (0.5)
Paid employment	5,469 (99.2)	6,570 (99.5)
Employment status, (%)		
Full-time	5,452 (99.3)	5,166 (79.9)
Part-time	29 (0.5)	1,180 (18.3)
Casual	12 (0.2)	116 (1.8)
Type of jobs, (%)		
Blue collar	3,132 (56.2)	2,442 (36.7)
White collar	2,437 (43.8)	4,204 (63.3)
ISCO-88 major groups*		
Legislators, senior officials, managers	287 (5.2)	54 (0.8)
Professionals	1,072 (19.3)	1,429 (21.5)
Technicians and associate professionals	638 (11.5)	480 (7.2)
Clerks	395 (7.1)	2,197 (33.1)
Service workers, shop, and sales workers	285 (5.1)	1,140 (17.2)
Skilled agriculture and fishery workers	353 (6.3)	163 (2.5)
Craft and related trade workers	1,349 (24.2)	253 (3.8)
Plant and machine operators and assemblers	791 (14.2)	375 (5.6)
Elementary occupations	243 (4.4)	404 (6.1)
Occupational settings		
Indoor only	3,045 (54.7)	6,123 (92.1)
Outdoor only	1,610 (28.9)	365 (5.5)
Indoor and outdoor	914 (16.4)	158 (2.4)
Occupational activities		
Moderate physical activity	1,000 (18.0)	2,822 (42.5)
Sitting/standing jobs	2,403 (43.2)	3,423 (51.5)
Vigorous physical activity	2,166 (38.9)	401 (6.03)

*People are classified in any of their three jobs reported.

More than half of the men worked in ‘blue-collar’ occupations (56%), and men were more likely than women to have a job involving vigorous physical activity (39% vs. 6%). Women predominantly worked indoors (92%), and just over half had roles that involved sitting and/or standing without physical activity (51%). Among men, 3% were current and 54% were former smokers, while among women, 2% were current and 31% were former smokers.

### Disability-free survival

Table 2 compares the primary outcome of disability-free survival among the ISCO-88 initial-digit occupational groups. A hazard ratio greater than 1.0 indicates a reduction in disability-free survival compared to all other participants. Using the statistical significance level of 0.005, a reduction in disability-free survival was observed for men in ‘elementary’ occupations (HR 1.29, 95% CI 1.07–1.56). This relationship remained after adjusting for additional confounders in the model, as shown in Supplementary Table S4.

**Table 2:** Association between disability-free survival and mortality in Australian healthy older adults with ISCO-88 major occupational groups.

ISCO-88 single-digit occupational groups†		Reduction in disability-free survival (DFS)			All-cause mortality		
		*n*!	HR (95% CI)	Value of p*	*n*!	HR (95% CI)	Value of *p*
*Group 1*	*Legislators, senior officials, and managers*	45	1.07 (0.82–1.42)	0.598	33	1.15 (0.86–1.56)	0.346
*Group 2*	*Professionals*	311	0.83 (0.75–1.02)	0.096	191	1.00 (0.87–1.15)	0.996
*Group 3*	*Technicians and associate professionals*	162	1.04 (0.87–1.24)	0.667	100	1.01 (0.8–1.29)	0.877
*Group 4*	*Clerks*	311	0.96 (0.84–1.09)	0.559	182	0.84 (0.70–1.00)	0.053
*Group 5*	*Service workers, shop and sales workers*	184	1.01 (0.87–1.19)	0.814	114	0.99 (0.80–1.23)	0.985
*Group 6*	*Skilled agriculture and fishery workers*	61	1.26 (1.04–1.55)	0.022	40	1.19 (0.88–1.62)	0.257
*Group 7*	*Craft and related trade workers*	248	0.96 (0.84–1.11)	0.626	156	0.87 (0.73–1.03)	0.112
*Group 8*	*Plant and machine operators and assemblers*	171	1.15 (0.98–1.35)	0.081	109	1.26 (1.03–1.56)	0.025
*Group 9*	*Elementary occupations*	109	**1.29 (1.07–1.56)**	**0.005**	75	1.12 (1.07–1.39)	0.029
	*Rate per 1,000 person-year*	18.1			12.03		

The HR were adjusted for age, sex, and smoking. !Number of exposed individuals with an outcome of DFS or mortality. †Participants not in the specific occupational category were used as a reference group. Individuals ever employed in one of these occupational groups were included (therefore, a single individual might have been included in more than one group). A value of *p* < 0.005 was considered statistically significant and presented as bold in the table.

A reduction in disability-free survival was also found among participants reporting jobs in occupational groupings defined by FINJEM as having a high accident risk (HR 1.35, 95% CI 1.15–1.61) or associated with an ‘adverse social climate’ at work (refers to communication, information flow, and cooperation; full definitions in Supplementary Table S2) (HR 1.86, 95% CI 1.11–1.97) (Table 3). There was no evidence of a reduction in disability-free survival among those whose past occupational history involved sedentary work, vigorous physical activity, work primarily outdoors, or a range of other occupational characteristics (Table 4).

**Table 3:** Association between disability-free survival and mortality in Australian healthy older adults and occupational factors.

Occupational factors from FINJEM*	Reduction in disability-free survival			All-cause mortality		
	*n*†	HR (95% CI)	Value of *p*‡	*n*†	HR (95% CI)	Value of *p*‡
Psychological and organizational stress factors						
*Challenge at work*	18	0.50 (0.49–1.1)	0.285	7	0.54 (0.32–0.89)	0.01
*Control possibilities at work*	17	1.19 (0.83–1.7)	0.332	8	1.41 (0.71–2.77)	0.325
*Perceived risks at work*	17	1.22 (0.85–1.74)	0.299	9	1.25 (0.89–1.73)	0.185
*Adverse social climate at work*	12	1.86 (1.11–1.97)	0.002	11	1.49 (0.8–2.78)	0.21
*Social demand at work*	24	0.67 (0.47–0.96)	0.028	17	0.77 (0.60–0.98)	0.039
*Supervisor support*	16	0.77 (0.35–1.73)	0.532	8	0.99 (0.99–1.01)	0.352
*Working time arrangement*	18	0.67 (0.36–1.27)	0.219	9	0.92 (0.50–1.72)	0.808
Physical factors						
*Ionizing radiation*	4	1.88 (0.54–6.52)	0.319	4	2.52 (1.15–5.54)	0.020
*Low-frequency magnetic fields*	35	0.82 (0.54–1.23)	0.349	20	0.97 (0.59–1.59)	0.92
*Noise*	11	0.65 (0.44–0.99)	0.044	8	0.75 (0.39–1.43)	0.384
*Ultraviolet radiation*	31	0.95 (0.68–1.33)	0.758	17	0.92 (0.51–1.65)	0.776
Ergonomic factors						
*High accident risk*	120	1.35 (1.15–1.61)	<0.001	72	1.29 (1.08–1.49)	0.028
*Inconvenient and difficult work postures*	65	1.14 (0.89–1.45)	0.303	40	1.2 (1.07–1.4)	0.033
*Manual handling*	69	0.94 (0.77–1.15)	0.569	44	0.96 (0.76–1.22)	0.692
*Perceived physical workload*	65	0.94 (0.72–1.23)	0.654	39	0.86 (0.60–1.23)	0.418
*Repetitive work movements*	86	1.04 (0.84–1.28)	0.711	54	0.97 (0.72–1.31)	0.864
*Sedentary work*	10	1.25 (0.79–1.95)	0.331	11	1.23 (0.64–2.39)	0.536
*Work with video display units*	96	0.92 (0.75–1.15)	0.481	59	0.99 (0.78–1.26)	0.923

The hazard ratios were adjusted for age, sex, and smoking. †Number of participants with outcome and exposure. ‡A value of *p* < 0.005 was considered statistically significant and presented as bold in the table. *Definitions of occupational factors are included in the online Supplementary material.

**Table 4:** Association between disability-free survival and mortality in Australian healthy older adults with occupational setting and activity.

Occupational factors	Reduction in disability-free survival			All-cause mortality		
	*n*†	HR (95% CI)	Value of p*	*n*†	HR (95% CI)	Value of p*
Occupational settings						
Men						
Indoor only	464	Ref.		321	Ref.	
Outdoor only	248	1.21 (1.03–1.42)	0.017	171	1.02 (0.83–1.24)	0.793
Indoor and outdoor	137	1.01 (0.84–1.22)	0.91	97	1.02 (0.82–1.25)	0.882
Women						
Indoor only	732	Ref.		392	Ref.	
Outdoor only	39	1.46 (1.08–1.95)	0.012	22	1.40 (1.07–2.02)	0.041
Indoor and outdoor	21	0.67 (0.46–1.02)	0.059	13	0.82 (0.51–1.31)	0.115
Occupational activities						
Men						
Moderate physical activity	166	Ref.		119	Ref.	
Sitting/standing jobs	355	1.01 (0.87–1.16)	0.945	246	0.93 (0.73–1.18)	0.486
Vigorous physical activity	328	1.38 (1.09–1.44)	0.027	224	1.17 (0.96–1.44)	0.11
Women						
Moderate physical activity	359	Ref.		194	Ref.	
Sitting/standing jobs	387	1.01 (0.88–1.18)	0.811	210	0.91 (0.75–1.12)	0.378
Vigorous physical activity	46	1.12 (0.82–1.53)	0.473	23	1.29 (1.07–1.85)	0.039

The hazard ratios were adjusted for age and smoking. †Number participants with exposure and endpoints. *A value of *p* < 0.005 was considered statistically significant.

### All-cause mortality

All-cause mortality was not significantly increased among any major occupational groups (Table 2) nor were specific risks identified as reaching the predefined level of statistical significance (*p* < 0.005; Table 3) or activity levels in the workplace or indoor/outdoor work locations (Table 4).

## Discussion

This large prospective survivor’s cohort study is the first to examine the association between occupational characteristics and disability-free survival in an older adult population in the post-retirement period. Disability-free survival is a surrogate measure of independent living which is particularly relevant in an aging population. The principal finding was a reduction in disability-free survival associated with the least skilled or ‘elementary’ occupations. These include cleaners, garbage collectors, and laborers in agriculture, fisheries, mining, construction, mining, and manufacturing.

Among the specific job characteristics, those with the major adverse influence on disability-free survival were working in an adverse social environment and work that involved a high accident risk. All of these are likely to be characteristics of ‘elementary’ occupations and to provide a potential explanation for the lower likelihood of healthy survival among these workers.

Of the ASPREE participants who died, 45% developed either dementia or persistent physical disability prior to death (17). However, all-cause mortality was not substantially different among the four-digit ISCO-88 occupation groups, including elementary occupations. This indicates that the significant reduction in disability-free survival among the elementary occupational groups is likely to have resulted from an increase in physical disability or dementia related to occupational history (18).

The relatively similar mortality among participants, including those working in elementary occupational groups contrasts with previous reports including the United States National Longitudinal Mortality Study (1) and the Whitehall study of United Kingdom civil servants (2). A report using Korean insurance claim data also found higher mortality among lower occupational categories and a study from Japan found a high mortality in elementary occupations with most of the mortality due to cancer (19, 20). As all participants in ASPREE were recruited as healthy, the analyses of mortality may be biased due to the selective exclusion of those most likely to die prematurely from occupational factors. Alternatively, it might reflect a declining social gradient within the Australian workforce or the impact of occupational health and safety standards introduced progressively over the working life of the ASPREE participants.

The results of this study add to a growing literature on the effect of occupational characteristics and exposures on other aspects of health in later life. These include a recent Global Burden of Disease study, which reported an increase in disability-adjusted life years attributable to several occupational factors (21). Several population-based cohorts have also reported links between physically demanding jobs, shift work, ergonomic and psychological stress factors, and all-cause mortality (4, 5, 7–9). The results of recent community-based cohort studies investigating the relationship between occupational factors and mortality are summarized in Supplementary Table S5. In particular, the association between heavy physical work and mortality, particularly in men has also been documented in other occupational studies (4, 6, 22).

Overall, this study suggests that some specific occupational characteristics may lead to adverse outcomes later in post-retirement age. Despite this, it is impossible to exclude the likelihood that the reduction in healthy survival reflects socioeconomic disadvantage, poor health behaviors, and the likelihood that individuals with pre-existing physical or cognitive limitations select elementary occupations. Smoking, in particular, was more common among individuals working in low-skilled labor-intensive occupations, but the influence of cigarette smoking was at least partially controlled in our analysis.

The public health significance of these findings arises from the substantial proportion of the workforce who undertake elementary and laboring occupations for a significant part of their working lives. Based on this analysis, these individuals may generate substantial additional costs for supportive care and social services in their older years. This emphasizes the need to determine more precisely whether job characteristics or other (e.g., lifestyle) characteristics lead to adverse health outcomes. Meanwhile, attention is needed to mitigate the potentially harmful effects of the negative attributes identified in this study.

This study has several strengths. For the first time, we investigated the association between occupational factors and disability-free survival and all-cause mortality in a sample from a healthy general population aged 70 years and older, in both men and women. The ASPREE study was subject to rigorous quality control, and all endpoints were actively screened, and verified by access to clinical records, which allowed the identification of the proximal cause of death. We used FINJEM, which is designed for a general population-based study. The advantage of using JEM is that it excludes recall bias and minimizes differential misclassification. In addition, the FINJEM period allows us to consider exposure at the time of employment. Finally, we adjusted for smoking as a confounder in the final models and performed a range of sensitivity analyzes to overcome the effect of residual confounding in the analyses.

Limitations included the restriction of occupational histories to the three longest-held jobs rather than a complete job history. Increased risks among women are likely to be underestimated as a result of their shorter duration in the workforce in this cohort (23). Alcohol intake is a potential confounder but was not included in the models because of the likely low validity of self-reports, its close correlation with smoking, and the very small number of individuals in this population declaring a high alcohol intake. FINJEM classifies exposures based on job titles only and cannot account for variations in characteristics of jobs with the same title. Being based on Finnish occupational data, the use of FINJEM to assess occupational factors, such as adverse social climate, might not necessarily reflect Australian working conditions.

It should be noted that the current investigation considered all Australian ASPREE participants, irrespective of their treatment allocation to either low-dose aspirin or placebo in the main intervention trial. The decision to include all Australian participants was made in light of the widespread use of aspirin in the population, with 7.2% of Australian participants being regular users prior to the trial (24). Nevertheless, sensitivity analyses demonstrated that adjusting for additional confounding factors had no impact on the overall effect estimates, as presented in Supplementary Table S4. A second analysis in which the results were presented adjusted for aspirin use in the trial allocation (data not shown) also found no discernible differences between the two groups.

In summary, this study reports the impact of occupational characteristics on disability-free survival among a large older initially healthy population recruited from the Australian community. Among the various ISCO-88 job categories, only those belonging to the least skilled ‘elementary’ occupations experienced a reduction in disability-free survival. Among the specific job characteristics, work in occupations with a high accident risk or in an adverse social climate were both associated with reduced disability-free survival, and potentially contributed to health disadvantage. All-cause mortality was not substantially increased among any of the occupational groupings or with any specific occupational characteristics. This may reflect survivor bias among individuals in this age group, especially those selected for their health at baseline.

## Data availability statement

The raw data supporting the conclusions of this article will be made available by the authors, without undue reservation.

## Ethics statement

The studies involving humans were approved by Monash University Ethics Committee (project numbers CF11/1100 and CF11/1935). The studies were conducted in accordance with the local legislation and institutional requirements. The participants provided their written informed consent to participate in this study.

## Author contributions

SA, GB, and JM conceived and designed the study. AO had significant involvement in data collection. KR and CT completed the ISCO-88 coding. SA conducted the cross work to Finnish-O codes and performed data analysis, interpretation, producing the initial draft of the manuscript. SA and JM approved the final version of the manuscript for publication and jointly took responsibility for the accuracy and integrity of all aspects of the research. All authors contributed to the article and approved the submitted version.
