# Supplementary material for: Occupational characteristics and disability-free survival after retirement age: an exploratory analysis from the ASPREE study
Source: Front Public Health. 2023 Dec 15;11:1191343. doi: 10.3389/fpubh.2023.1191343 (PMC10773837; doi:10.3389/fpubh.2023.1191343)
Supplement: Supplementary file 1 [file Data_Sheet_1.docx]

# Supplementary material

Occupational characteristics and disability-free survival after retirement age: an exploratory analysis from ASPREE study

**Methods**

## Occupational data coding

Each ISCO-88 code was aligned with Finnish O-codes to assign the occupational exposures. Finnish job titles were searched during this process, and relevant codes were matched and converted to O-codes. In each stage, coders were guided by investigators with experience in job coding, with differences resolved by consensus. In the final stage, the investigators reviewed all ISCO-88 and O-codes to ensure the comparability of job titles in the entire cohort. Participants were considered to be exposed if the probability of exposure was >25% (1). Time periods from FINJEM were used based on ALSOP participants’ working life, which was 1945-59, 1960-84, 1985-94, 1995-97, 1998-00, 2001-03, 2004-06, and 2007-09.

## Occupational coding and ISCO-88 job classification

***International Standard Classification of Occupations (ISCO-88)*** of International Labour Organization (ILO) provides a systematic classification and aggregation of occupational information covering the entire working population using 4-digit codes (2). ISCO-88 is a revision of ISCO-68 version, which it supersedes, and it is one of the standard skills based occupational models of ILO. The design of ISCO-88 was based on two major components: (I) Job defined as a set of task or duties executed or meant to be executed by a person, and (II) skill- defined as the ability to carry out the tasks and duties of a given job. On the basis of skill and educational categories from the International Standard Classification of Education (ISCED), four subjective skill levels were defined. Based on the skill levels of technical criterion, codes were established that allow a hierarchical occupational classification that consists of ten major groups that further subdivided into 28 sub-major groups, 116 minor groups, and 390-unit groups (Table S1). The first digit of the code represents the major group that includes all possible occupations from the general population. Sub-major groups are two-digit codes, where the 28 sub-major groups fall. Minor groups are identified by three-digit codes, where the first two digits indicate the sub-major group where the minor group falls. These minor groups consider workers’ tasks, kinds of goods and service produced and differ according to the technical level required. The 390-unit groups represent more detailed groups of occupations that are similar to the job characteristics. They are identified by codes of four-digits. The cohort population was defined as having at least one of the job titles from the ISCO-88 job code.

## Covariates

Covariates were identified using Directed Acyclic Graph (DAG) from DAGitty software. The DAG model suggested age, sex, smoking, and education as potential confounder. Lifetime smoking of the participants was categorised as ever and never-smokers.

**Table S 1** ISCO-88 occupational classifications (2)

| **Major groups** | **Summary of major groups** | **Sub-major groups** | **Minor groups** | **Unit groups** |
| --- | --- | --- | --- | --- |
| *1. Legislators, senior officials, and managers* | Determiners and formulators of government policies, laws, and public regulations; managers of large and small companies and organizations | 4 | 8 | 33 |
| *2. Professionals* | Professionals in engineering, physical sciences, computer science, health, life sciences, social sciences, humanities, education and legal and financial affairs | 4 | 18 | 55 |
| *3. Technicians and associate professionals* | Technicians in engineering, physical sciences, data management, health, life sciences, social sciences, humanities, education, administration, ship and aircraft controllers, government officials, business agents, electronic equipment operators | 4 | 21 | 73 |
| *4. Clerks* | Those who organize, store, compute and retrieve information for secretarial duties, word processing, office machines or computing numerical data, receptionists, telephonists. | 2 | 7 | 23 |
| *5. Service workers and shop and market sales workers* | Providers of protective services, sales workers, housekeepers, caterers and personal care | 2 | 9 | 23 |
| *6. Skilled agricultural and fishery workers* | Producers of farm, forestry and fishery products including growing crops, breeding or hunting animals, catching or cultivating fish, conserving, exploiting forests, selling agriculture or fishery products | 2 | 6 | 17 |
| *7. Craft and related trade workers* | Extractors of raw materials, building construction and finishing workers, mechanics, metal workers, printers, food processors, wood, textile and leather workers and makers of various handicraft goods | 4 | 16 | 70 |
| *8. Plant and machine operators and assemblers* | Operators and monitors of large scale, highly automated, industrial machinery and equipment | 3 | 20 | 70 |
| *9. Elementary occupations* | Users of hand-held tools, selling goods in streets, doorkeeping, cleaning, washing, pressing, labourers in mining, agriculture, fishery, construction, and manufacturing | 3 | 10 | 25 |
| *10.* | Armed forces | 1 | 1 | 1 |
| Totals |  | 28 | 116 | 390 |

**Table S2: Definitions for psychological, physical and ergonomic terms used in FINJEM** (3)

| Psychological stress factors |
| --- |
| **Challenge at work:** The variability, meaningfulness, and interesting tasks at work; subjective perception. All occupations are assessed on the basis of responses to 4 questions: My work is interesting (completely false - completely true). How often can you do such things at work that you are genuinely interested in (never - nearly all the time)? Is your work monotonous or variable (very monotonous - full of variety)? Do you find your present work important and significant (not at all significant - very important and significant))? (Quality of Working Life Survey 1990, Finland) |
| **Psychological workload:** The demand to work under tight schedules and time pressure, and to adjust conflicting demands from others; subjective perception. All occupations are assessed on the basis of responses to 4 questions: Do tight schedules and time pressure burden you at work (not at all - very much)? Does your work require the ability to fit together conflicting demands from different sources (not at all - very much)? Does your work require the ability to take care of several matters simultaneously? How often do you work under such pressure that you have no time to talk or think about anything else than work (never - nearly all the time)? (Quality or Working Life Survey 1990) |
| **Control possibilities at work:** The possibilities for independence and to influence one's working pace and methods; subjective perception. All occupations are assessed on the basis of responses to 5 questions: I can work independently (completely false - completely true). Do you have influence on the order in which you do your work tasks (not at all - very much)? Do you have influence on your working pace (not at all - very much)? Do you have influence on your working methods (not at all - very much)? How often can you set your own working pace (never - nearly all the time)? (Quality of Working Life Survey 1990) |
| **Perceived risks at work:** The risk to meet with or cause an accident or damage property due to an error in the normal course of action; subjective perception. All occupations are assessed on the basis of responses to 3 questions: Do you experience following risks at your work (not experienced as a risk - experienced as an obvious risk): risk of an accident, risk of causing an accident to someone else, risk of damaging valuable property or work results. (Quality of Working Life Survey 1990) |
| **Adverse social climate at work:** The degree of open communication, information flow, and cooperation; subjective perception. All occupations are assessed on the basis of responses to 4 questions: At my workplace there is an open atmosphere and feeling of team spirit (completely disagrees - completely agrees). Gossip and envy occur at my workplace (completely agrees - completely disagrees). There is open communication at my workplace (completely disagrees - completely agrees). Most people at my workplace try above all to keep up good interpersonal relationships (completely disagrees - completely agrees). (Quality of Working Life Survey 1990) |
| **Social demand at work:** The demand to communicate with other people than workmates, to serve and understand other people; subjective perception. All occupations are assessed on the basis of responses to 3 questions: Does your work require willingness to be of service (not at all - very much)? Does your work require ability to understand other people's problems (not at all - very much)? How often do you come into contact with people other than workmates at work (never - nearly all the time)? (Quality of Working Life Survey 1990) |
| **Supervisor support:** The encouragement, trust, and support from the supervisor, communications with the supervisor; subjective perception. All occupations are assessed on the basis of responses to 5 questions: Do you get support and encouragement from your supervisor if you find your work difficult (never - always)? My supervisor gives me support and encourages me (completely disagrees - completely agrees). My supervisor is an inspirer (completely disagrees - completely agrees). My supervisor often discusses matters with us (completely disagrees - completely agrees). My supervisor trusts his/her subordinates (completely disagrees - completely agrees). (Quality of Working Life Survey 1990) |
| **Working time arrangement:** Exposure to different working time arrangements, with emphasis on night work; the employee's reply. All occupations are assessed on the basis of responses to the question 'How is your working time arranged?' (Quality of Working Life Survey 1990). Occupations in which the proportion of exposed respondents exceeds 5 % are included. |
| Physical factors: |
| **Ionizing radiation:** Occupational exposure to ionizing radiation (energy >12.4 electron volts, eg, x-rays, gamma rays, cosmic radiation, alfa and beta particles) exceeding 0,2 mSv per year. [The annual equivalent radiation dose in millisieverts; equivalent dose (Sv) = absorbed dose (Gy) x quality factor Q (depends on the type of radiation); 1 Sv = 100 rem]. Occupations or industries where mean exposure may exceed exposure limits of 20 mSv/5y or 50 mSv/y are monitored by Finnish Institute of Radiation Safety. In addition, air carrier personnel may be exposed to levels exceeding 0.2 mSv/y. Other occupations were considered as occupationally unexposed. |
| **Low frequency magnetic fields:** Occupational exposure to low frequency ( <1 kHz ) magnetic fields over 0.5 µT. [mean daily magnetic flux density in microTeslas]. At least 5% of the occupation exposed to mean daily magnetic field exceeding 0.5 µT at any time in 1945-95. |
| **Noise:** Perceived occupational exposure to noise levels higher than 80 dB and higher than 85 dB [The annual median equivalent sound pressure level in decibels (weighted average)]. The perceived exposure of at least 25% of work time to noise level where normal speech is not audible and also measured from occupations where noise-induced hearing loss occurred in Finland. |
| **Ultraviolet radiation:** occupational exposure to ultraviolet radiation from the sun or artificial sources over 50 J/m2 [daily mean of biologically weighted effective energy density in joule per meter squared; 200 J/m2 = 1 MED]. Possibly at least 5% of the occupation exposed to a daily mean UV radiation energy exceeding 50 J/m2 at any time in 1945-95. |
| Ergonomic factors: |
| **High accident risk:** The work method or environment contains factors which are perceived as highly hazardous or the reported amount of incidence of accidents is high during last 12 months. |
| **Inconvenient and difficult work posture:** Working in postures, where the worker has to perform his/her tasks in bent, twisted, squatted or other difficult and strenuous postures, is an essential feature of normal work tasks as measured by subjective ratings. |
| **Manual handling of burdens:** Manual handling, which consists of lifting and carrying of heavy burdens, is an essential feature of the everyday work tasks. Measured by subjective ratings or observations. |
| **Perceived physical workload:** Perceived physical workload consists of tasks where the whole body is exerted by dynamic muscular work. The perceived load is measured by questionnaires. |
| **Repetitive work movements:** The work consists of work movements which are continuously repeated as such and experienced as hazardous by the worker. They are most common in hands, wrists and fingers like e.g., in tapping, screwing, assembly. The part of the body is not defined in all the studies used. |
| **Sedentary work**: Sedentary work consists of work done in seated posture, measured by subjective rating in questionnaire surveys. |
| **Work with video display units**: Occupational exposure due to working with video display units (microcomputer, computer terminal, word processor, other device used for controlling a production process). |

**Table S 3** ASPREE health measures and definitions (4)

| ***Annual health measurements*** |
| --- |
| - Demographic and lifestyle factors |
| - Weight, height, smoking status-current, former, never |
| - Depression screening questionnaire |
| - LIFE disability questionnaire including the Katz basic Activities of Daily Living (including walking across a room, bathing, dressing, transferring from a bed or chair, using the toilet, and eating); participants selected one of the following options for completing these tasks with ‘no difficulty’, ‘a little difficulty’ ‘some difficulty’, ‘a lot of difficulty’ or ‘unable to perform independently’; and, as a check, answered whether assistance from another person was required to complete |
| - Other clinical events |
| ***6-month phone calls*** |
| - confirmation of living circumstances |
| - administration of the Katz basic Activities of Daily Living |
| - clinical and adverse events report |
| ***Biennial health measures*** |
| - neurocognitive assessments |
| - physical function tests |

**Table S 4** Sensitivity analysis adjusted for additional confounders, alcohol intake, BMI and disease status for the association of DFS and mortality with ISCO-88 major occupational groups.

| **ISCO-88 major occupational groups** | | **Reduction in Disability free survival** | | | **All-cause mortality** | | |
| --- | --- | --- | --- | --- | --- | --- | --- |
|  |  | **n**† | **HR (95% CI)** | **p-value** | **n**† | **HR (95% CI)** | **p-value** |
| ***Group 1*** | ***Legislator, senior officials, managers*** | 269 | 1.02 (0.90-1.17) | 0.668 | 187 | 1.07 (0.91-1.26) | 0.388 |
| ***Group 2*** | ***Professionals*** | 392 | 0.90 (0.80-1.02) | 0.095 | 240 | 1.01 (0.87-1.16) | 0.902 |
| ***Group 3*** | ***Technicians and associate professionals*** | 262 | 0.89 (0.78-1.01) | 0.074 | 150 | 0.96 (0.82-1.14) | 0.663 |
| ***Group 4*** | ***Clerks*** | 427 | 0.95 (0.84-1.07) | 0.395 | 250 | 0.87 (0.75-1.02) | 0.085 |
| ***Group 5*** | ***Service workers, shop and sales,*** | 264 | 1.02 (0.89-1.16) | 0.781 | 148 | 0.92 (0.77-1.10) | 0.382 |
| ***Group 6*** | ***Skilled agriculture and fishery workers*** | 112 | 1.24 (1.02-1.50) | 0.031 | 67 | 1.23 (1.09-1.50) | 0.008 |
| ***Group 7*** | ***Craft and related trade workers*** | 275 | 0.96 (0.84-1.10) | 0.548 | 177 | 0.91 (0.77-1.07) | 0.293 |
| ***Group 8*** | ***Plant and machine operators and assemblers*** | 182 | 1.15 (0.99-1.34) | 0.064 | 117 | **1.34 (1.11-1.63)** | **0.003** |
| ***Group 9*** | ***Elementary occupations*** | 100 | **1.26 (1.09-1.51)** | **0.003** | 68 | 1.12 (1.09-1.40) | 0.009 |

The models were adjusted for age, sex, smoking, education, alcohol intake, BMI and disease status (hypertension) Participants not in the specific occupational category were used as a reference category.

† Number of participants with the exposure and the endpoint.

*p-value <0.05 were considered as statistically significant and presented as bold in the table.

**Table S 5** Population-based cohort studies investigated the association between occupational factors with mortality.

| **Author, year** | **Country and cohort** | **Sample size** | **Duration of follow-up (years)** | **Age of cohort (years)** | **Method of exposure assessment** | **Outcome data source** | **Summary of the studies** | **Confounders adjusted** |
| --- | --- | --- | --- | --- | --- | --- | --- | --- |
| **All-cause mortality** | | | | | | | | |
| Niedhammer, I. et al. 2021(5) | France, STRESSJEM data linkage cohort | 1496332 | 26 | 27 | STRESSJEM | National mortality data | Psychological work exposures increase the risk of all-cause mortality | Age as time scale variable, calendar time, biomechanical, physical, chemical and biological exposure were adjusted. |
| Niedhammer, I. et al. 2022(6) | France, STRESSJEM data linkage cohort | 1496332 | 26 | 27 | STRESSJEM | National mortality data | Shift work increase the risk of all-cause, cancer and other mortality | Age as time scale variable, calendar time, biomechanical, physical, chemical and biological exposure were adjusted. |
| Martinez Gomez et al. 2021(7) | USA, NIH-AARP Diet and Health study | 322126 | 1 | 50-71 | Questionnaire survey | NDI | Physically demanding jobs were associated with high mortality in men and women in unadjusted model but not in adjusted model. | Age, sex, education, race, smoking, deprivation index, healthy eating index, energy index, alcohol |
| Sakaue et al. 2020(8) | Japan, Tanushimaru community | 1680 | 15.9 | 40-75 | Questionnaire survey | NDI, hospital charts, medical records | Higher levels of physical activity reduced mortality, and that longer occupational sitting time increased mortality. | Age and sex |
| Tanaka et al. 2020(9) | Japan | 24863422 | 5 | 25-64 | National Census Data | NVS and MHLW | High all-cause and cardiovascular mortality in agriculture, forestry, fishing, mining, manufacturing, health and transport workers | Age-standardised mortality for men |
| Ervasti et al. 2019(10) | Finland, HHS cohort | 18387 | 9 | 48 | Questionnaire responses and JEM | Statistics Finland registers and Finnish centre for pension | Heavy physical work, lifting, carrying was associated with increased risk of death, mostly in men | Age, sex, education and chronic disease |
| Tanaka et al. 2019(11) | Japan, South Korea, Finland, Denmark, UK, France, Switzerland, Italy, Estonia and Lithuania | 293370858 | 15 | 35-64 | National register-based data | Mortality data matched with national register | Manual workers had higher mortality than upper non-manual workers. In the most recent time-period, upper non-manual workers had higher mortality than manual workers in Japan and South Korea. | Age |
| Mikkola et al. 2019(12) | Finland, HBS | 9935 | 26 | 45-57 | JEM | National Death Register | Men exposed to heavy physical work at their late career have an increased risk of all-cause and cardiovascular mortality as compared with men in physically light occupations. | Age, education, income |
| Lee et al. 2016(13) | Korea, NEIP | 8762340 | 5 | <15 to <60 | Insurance claim data | KNSO | Certain occupations such as agriculture, forestry and fishery workers had highest mortality risk followed by elementary occupations and plant, machine operators | Age, sex |
| Stamatakis et al. 2013(14) | UK and Scotland, Pooled sample HSE and SHS cohorts | 5788 men and 5380 women | 12.9 | 50-60 | Questionnaire survey | NHS | Sitting occupations are linked to increased risk for all-cause and cancer mortality in women but no association for cardiovascular mortality in men and women | Age, waist circumference, self-reported general health, alcohol intake, cigarette smoking, psychological health, non-occupational physical activity, prevalent cardiovascular disease, and prevalent cancer at baseline |
| Charles et al. 2010(15) | USA, HHP | 7540 | 3 | <58 | Self-reported questionnaire and expert assessment | Surveillance system, hospital records, and death certificates. | Occupational exposure to pesticides, metals, and solvents obtained during middle age was independently associated with increased mortality from all causes, cancer, and in specific cases, circulatory diseases. | Education, smoking status, triglycerides, physical activity, alcohol intake, and systolic blood pressure. |
| Johnson et al. 1999(16) | US, NLMS | 215165 male and  164693 females | 10 | 25-64 | Census data | NDI | Less skilled, labour-intensive occupations, taxi drivers, cooks, longshoremen and transportation operatives were high risk or mortality | Stratified by sex, adjusted for income, education and household size |

MHLW=Ministry of Health, Labour and Welfare; NDI=National Death Index; NVS=National Vital Statistics

Neidhammer et al. study linked two data sources, job history from 1976-2002 from French National working population of employees and French National

# References

1. Abramson MJ, Murambadoro T, Alif SM, Benke GP, Dharmage SC, Glaspole I, Hopkins P, Hoy RF, Klebe S, Moodley Y, Rawson S, Reynolds PN, Wolfe R, Corte TJ, Walters EH. Occupational and environmental risk factors for idiopathic pulmonary fibrosis in Australia: case-control study. *Thorax* 2020; 75: 864-869.

2. International Labour Organization. International Standard Classification of Occupations (ISCO) 18 September 2004. https://www.ilo.org/public/english/bureau/stat/isco/isco88/major.htm and https://www.ilo.org/public/english/bureau/stat/isco/isco88/alpha.htm (Accessed 7 November 2023)

3. Kauppinen T, Toikkanen J, Pukkala E: From cross-tabulations to multipurpose exposure information systems: a new job-exposure matrix. Am J Ind Med 1998, 33:409–417

4. McNeil JJ, Woods RL, Nelson MR, Reid CM, Kirpach B, Wolfe R, Storey E, Shah RC, Lockery JE, Tonkin AM, Newman AB, Williamson JD, Margolis KL, Ernst ME, Abhayaratna WP, Stocks N, Fitzgerald SM, Orchard SG, Trevaks RE, Beilin LJ, Donnan GA, Gibbs P, Johnston CI, Ryan J, Radziszewska B, Grimm R, Murray AM. Effect of Aspirin on Disability-free Survival in the Healthy Elderly. *N Engl J Med* 2018; 379: 1499-1508.

5. Niedhammer I, Milner A, Coutrot T, Geoffroy-Perez B, LaMontagne AD, Chastang JF. Psychosocial Work Factors of the Job Strain Model and All-Cause Mortality: The STRESSJEM Prospective Cohort Study. *Psychosom Med* 2021; 83: 62-70.

6. Niedhammer I, Coutrot T, Geoffroy-Perez B, Chastang JF. Shift and Night Work and All-Cause and Cause-Specific Mortality: Prospective Results From the STRESSJEM Study. *J Biol Rhythms* 2022; 37: 249-259.

7. Martinez Gomez D, Coenen P, Celis-Morales C, Mota J, Rodriguez-Artalejo F, Matthews C, Saint-Maurice PF. Lifetime high occupational physical activity and total and cause-specific mortality among 320 000 adults in the NIH-AARP study: a cohort study. *Occup Environ Med* 2022; 79: 147-154.

8. Sakaue A, Adachi H, Enomoto M, Fukami A, Kumagai E, Nakamura S, Nohara Y, Kono S, Nakao E, Morikawa N, Tsuru T, Hamamura H, Yoshida N, Fukumoto Y. Association between physical activity, occupational sitting time and mortality in a general population: An 18-year prospective survey in Tanushimaru, Japan. *Eur J Prev Cardiol* 2020; 27: 758-766.

9. Tanaka H, Tanaka T, Wada K. Mortality by occupation and industry among Japanese men in the 2015 fiscal year. *Environ Health Prev Med* 2020; 25: 37.

10. Ervasti J, Pietiläinen O, Rahkonen O, Lahelma E, Kouvonen A, Lallukka T, Mänty M. Long-term exposure to heavy physical work, disability pension due to musculoskeletal disorders and all-cause mortality: 20-year follow-up-introducing Helsinki Health Study job exposure matrix. *Int Arch Occup Environ Health* 2019; 92: 337-345.

11. Tanaka H, Nusselder WJ, Bopp M, Brønnum-Hansen H, Kalediene R, Lee JS, Leinsalu M, Martikainen P, Menvielle G, Kobayashi Y. Mortality inequalities by occupational class among men in Japan, South Korea and eight European countries: a national register-based study, 1990–2015. *J Epidemiol Community Health* 2019; 73: 750-758.

12. Mikkola TM, von Bonsdorff MB, Salonen MK, Kautiainen H, Ala-Mursula L, Solovieva S, Viikari-Juntura E, Eriksson JG. Physical heaviness of work and sitting at work as predictors of mortality: a 26-year follow-up of the Helsinki Birth Cohort Study. *BMJ Open* 2019; 9: e026280.

13. Lee H-E, Kim H-R, Chung YK, Kang S-K, Kim E-A. Mortality rates by occupation in Korea: a nationwide, 13-year follow-up study. *Occup Environ Med* 2016; 73: 329-335.

14. Stamatakis E, Chau JY, Pedisic Z, Bauman A, Macniven R, Coombs N, Hamer M. Are sitting occupations associated with increased all-cause, cancer, and cardiovascular disease mortality risk? A pooled analysis of seven British population cohorts. *PLoS One* 2013; 8: e73753.

15. Charles LE, Burchfiel CM, Fekedulegn D, Gu JK, Petrovitch H, Sanderson WT, Masaki K, Rodriguez BL, Andrew ME, Ross GW. Occupational exposure to pesticides, metals, and solvents: the impact on mortality rates in the Honolulu Heart Program. *Work* 2010; 37: 205-215.

16. Johnson NJ, Sorlie PD, Backlund E. The impact of specific occupation on mortality in the US National Longitudinal Mortality Study. *Demography* 1999; 36: 355-367.
